# Virtual reality exposure therapy for adolescents with fear of public speaking: a non-randomized feasibility and pilot study

**DOI:** 10.1186/s13034-019-0307-y

**Published:** 2019-12-27

**Authors:** Smiti Kahlon, Philip Lindner, Tine Nordgreen

**Affiliations:** 1 Division of Psychiatry, Haukeland University Hospital, Haukelandsbakken 15, 5009 Bergen, Norway; 2 Department of Psychology, Stockholm University, Stockholm, Sweden; 3 Centre for Psychiatry Research, Department of Clinical Neuroscience, Karolinska Institutet & Stockholm Health Care Services, Stockholm County Council, Stockholm, Sweden; 4 Department of Clinical Psychology, Faculty of Psychology, University of Bergen, Bergen, Norway

**Keywords:** Virtual reality, Virtual reality exposure therapy, Public Speaking Anxiety, Cognitive behavior therapy, Adolescents, Inhibitory learning

## Abstract

**Background:**

Public Speaking Anxiety (PSA) is a common anxiety with onset in adolescence and early adulthood. With the advent of consumer virtual reality (VR) technology, VR-delivered exposure therapy is now a scalable and practical treatment option and has previously been shown to be efficacious with adults. In this non-randomized feasibility and pilot trial, we explore the effect of one-session (90 min) VR-delivered exposure therapy for adolescents (aged 13–16) with PSA.

**Methods:**

A total of 27 adolescents were recruited from Norwegian high schools and completed self-report measures of PSA twice prior to treatment, 1 week after treatment, and at 1 and 3 month follow-up. Heart rate was recorded during the treatment session. A low-cost head-mounted VR display with a custom-built VR stimuli material depicting a cultural and age appropriate classroom and audience were used when a series of speech (exposure exercises) were performed.

**Results:**

Linear mixed effects model revealed a significant decrease in PSA symptoms (Cohen’s *d* = 1.53) pre-post treatment, and improvements were maintained at follow-ups. Physiological data revealed a small increase in heart rate during exposure tasks. Based on feedback from the adolescents, the feasibility of the intervention was increased during the trial.

**Conclusions:**

The results show that low-cost, consumer VR hardware can be used to deliver efficacious treatment for PSA in adolescents, in a feasible one-session format.

## Background

One out of three report anxiety symptoms when giving a speech in front of others [1], referred to as Public Speaking Anxiety (PSA). The most common fears in performance situations among individuals with PSA include showing signs of anxiety symptoms like shaking or trembling, the mind going blank while presenting, saying something stupid or not being able to continue to talk [2]. PSA is a distinct subgroup of the wider clinical presentation of social anxiety disorder (SAD) [3]. SAD is defined as the fear of negative evaluation of others in social situations, followed by feeling embarrassed or humiliated [4]. SAD is one of the most common psychiatric disorders with a life time prevalence of 13.7% in the Norwegian general population [5] and 4.0% across all countries [6]. The majority of the individuals with SAD report anxiety in performance situations [2]. SAD has an onset in adolescence with a mean age onset at 15 years [2, 7]. Over 80% of the individuals with SAD do not receive any treatment, and the mean age of first treatment is at 27 years [2].

The literature describes two subgroups of adult SAD: those with both interaction and performance anxiety (generalized SAD) and those with only performance anxiety [3], with PSA as the most common symptom in both adolescents [8] and adults [9]. Congruently, the novel DSM-5 [4] revised its specifier of SAD to include a “performance only” subgroup, distinct in terms of etiology, age at onset, physiological response, and treatment response [10].

Untreated PSA may cause further impairment in adulthood and around 50% of adolescents [11] and adults [3] with PSA develop generalized social anxiety [7]. Studies have shown that treating the specific subtype of PSA reduce the overall level of generalized social anxiety [12–14] and by providing a treatment intervention targeting adolescents, one might be able to reduce the societal and personal costs of the disease.

There is strong evidence supporting cognitive behavioral therapy (CBT) with exposure as the treatment of choice in treating PSA and SAD [15, 16]. However, conducting in-session exposure exercises for PSA has historically been unpractical or outright infeasible since this would require access to and control over an audience. This is in contrast with treatments of other anxiety disorders that rely on in-session exposure therapy, e.g. animal phobias and other specific phobias that are highly efficacious [17–20]. Virtual reality (VR) technology can resolve this issue by creating the illusion of being present in front of a realistic virtual audience. This is achieved by wearing a headset with dual displays that cover the eyes and simulates depth perception, the displayed content of which is interactive to head movement to give the illusion of being able to look around the virtual world [21]. By creating an animated virtual audience and presenting the feared stimuli to the patient, VR Exposure Therapy (VRET) for PSA is an attractive treatment method since it provides a convenient way doing in-session exposure with immediate access to controllable fear stimuli. Importantly, virtual audiences are sufficient to elicit a fear response [22], the basis of exposure therapy, and several randomized controlled trials of VRET for PSA have shown good results [23–26].

Until recently however, VR equipment was expensive, inaccessible, and required a high degree of technical competence to develop for and use. Lindner et al. [26] was the first to investigate whether consumer VR hardware and software can be used to conduct in-session exposure therapy with a therapist. One study has showed that relevant VR-stimuli do provoke distress in socially anxious youth [27]. To our knowledge, there have been no intervention studies on VRET for adolescents with PSA. In the current, non-randomized feasibility and pilot study, we investigate the feasibility of adapting the 3-h single session VRET protocol for PSA examined in Lindner et al. [26] into a 90-min single session for use with adolescents, and examine whether the effect size is similar, explore possible moderators of treatment effects, and physiological response to the VR scenarios.

## Methods

### Design

This study is a non-randomized feasibility and pilot study with pre, post and 1- and 3-month follow-ups. Reporting follows the CONSORT guidelines for pilot/feasibility trials.

### Ethics

The study received ethical approval from the Norwegian Regional Ethical Committee (REK 2017-1521). Written informed consent was obtained from the parents of adolescents at the training session.

### Procedure

A total of *N* = 27 adolescents were included in the feasibility and pilot study. Recruitment was done in two periods: spring 2018 and autumn 2018. Information about the study was given in classrooms at two high schools (8th to 10th grade). In addition, written information with a link to the study website was distributed to all students and parents through mail, as well as school health services and head masters at the schools in Bergen, Norway, and through Facebook. Interested participants accessed the study website and completed the online screening, including Public Speaking Anxiety Scale (PSAS; [28]) and Social Interaction Anxiety Scale (SIAS; [29]). In order to be included, the adolescents had to be between 13 and 16 years old, confirm that they were afraid of speaking in public, had to report symptoms of PSA on the PSAS (observed range: 46–73, possible range 17–85) and functional impairment due to PSA. Due to the lack of international established cut-offs, no threshold level for PSAS was applied. Exclusion criteria, assessed during the same phone call, were: ongoing psychotherapy, use of benzodiazepines, and lack of stereoscopic vision that would impair the VR experience. After the initial screening eligible participants were contacted by phone to schedule a date for the training session.

**Fig. 1:**
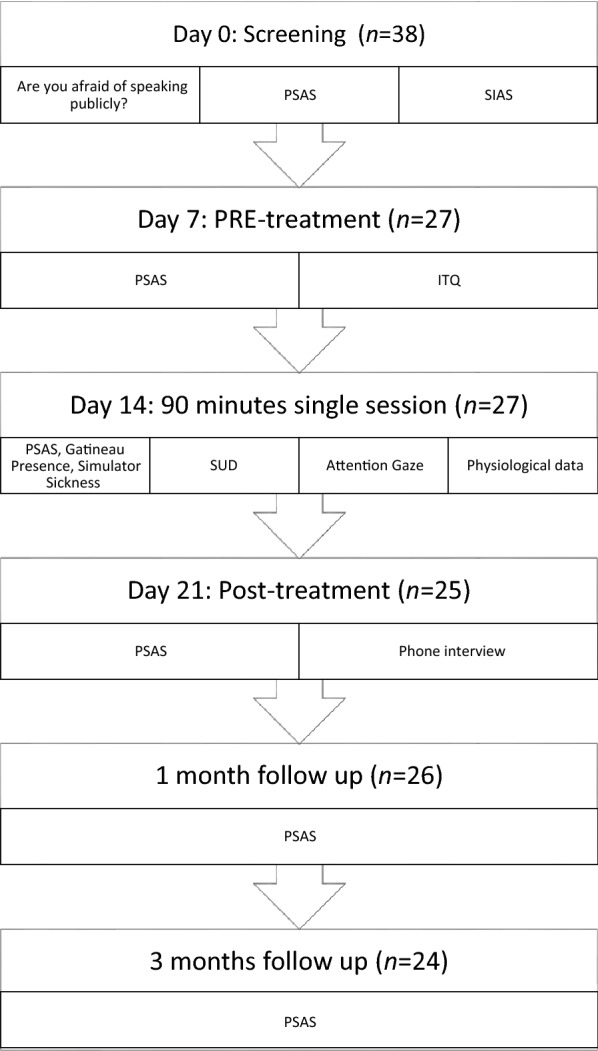
Study flowchart

Participants met for a single 90-min training session approximately 1 week after completing the online pretreatment questionnaires. Informed consent from parents was obtained before the exposure session. Post-treatment questionnaires were distributed 1 week after the training session. In addition, follow-up questionnaires were distributed one and 3 months after completing the treatment session, in order to evaluate the long-term effects of the treatment. See Fig. 1 for study flowchart. Participants who completed the training session and responded to the questionnaires received a gift certificate of 200 NOK (approximately 20 Euros) when completing the 1-month follow-up assessment.

### Intervention

#### VR scenario

**Fig. 2:**
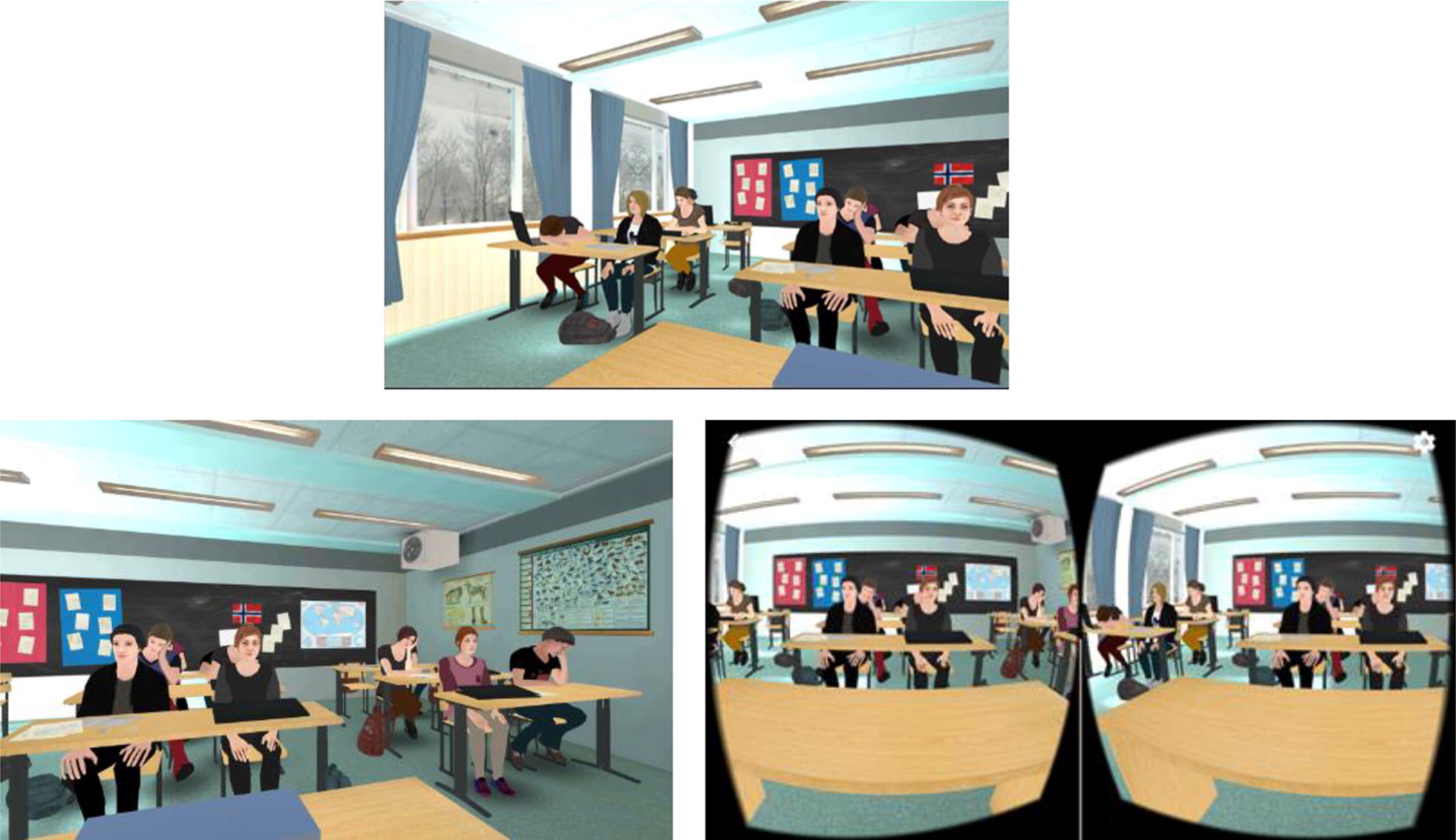
Screenshots from the VR application

A custom-built VR stimuli material depicting a typical Norwegian classroom and an age-appropriate audience was developed by Attensi AS, a professional IT developer. The scenario was inspired by five culturally and age-appropriate actual Norwegian classrooms and adjusted after feedback from testing by four adolescents. The classroom featured ten virtual avatars, depicted to be in the age range of 13–16, sitting at their desks, with minor body animations and gaze directed at the user situated at the front of the class. An empty classroom and a lobby were also available, with each exposure exercise beginning with the participant selecting to enter the full classroom. See Fig. 2 for screenshots. An Apple iPhone 7 and a high-end Cardboard-type VR headset (costing the equivalent of 60 USD) was used for stimuli presentation. The application automatically logged all user behaviors (e.g. entering and exiting the full classroom) with timestamps.

#### Treatment protocol

The treatment protocol was adapted from a recent VRET protocol for PSA [26], and tailored for use with adolescents. This protocol in turn was based on Öst’s [20] one-session treatment for specific phobias, with speech (exposure) exercises (e.g. count down from 60, say words beginning with the letter P, improvised speeches about everyday matters), partly drawn from a one-session in vivo exposure protocol for PSA [12]. The session was therapist-led and consisted of three parts: brief psychoeducation (approx. 15 min), followed by exposure tasks (approx. 60 min) and ending with a summary and introduction to active maintenance (approx. 15 min). The therapeutic goal was to promote inhibitory learning [30] by exposing participants to speech scenarios designed to test their idiosyncratic catastrophic beliefs about speech performance and physiological reaction. The psychoeducation consisted of a short introduction to the CBT model of anxiety with some VR-adjustments. A simple functional analysis of their PSA was performed and the treatment rationale was explained.

The treatment protocol included seven tasks with varying levels of difficulty that lasted 1 to 2 min each with no or little preparation time. Adolescents wore the VR device only during the actual exposure tasks in order to avoid habituation to the virtual environment in itself, outside an exposure context. Participants were first instructed to enter a neutral empty classroom to make sure that the VR device was correctly configured. The adolescents were then exposed to a virtual classroom with an animated audience. Each task required entering and leaving the virtual classroom from a lobby, with discussions with the therapist between the tasks. Prior to each exposure task, the therapist would give instructions and noting their catastrophic beliefs and expectancy rating on a scale from 0 to 100. The therapist recorded maximum and minimum subjective units of distress (SUD; [31]) on a 0–100 scale, where 0 corresponds to no distress at all and 100 is the worst possible, as reported by the participant directly after each exposure task. SUDS [31] is a useful tool by showing the participants how their level of anxiety reduces throughout the exposure tasks. Immediately after the exposure task, the adolescent would evaluate the performance together with the therapist and rate the subjective level of discomfort experienced. The therapist and adolescent then listened to an audio recording of the task, with the adolescent using mental imagery to cast themselves in the role of an audience member in the same scenario listening to the speech as played back. Participants were then asked to once again quality rate the performance after listening and discuss with the therapist about discrepancies before continuing to the next exposure task. This procedure was then repeated for each task.

After completing the exposure tasks, the adolescent and the therapist had a short summary discussion before introducing them how to practice exposure in real life. Main points were summed up in a folder, which was given to the adolescent at the end of the session. The duration of the session was reduced from an original 3 h [26] to approximately 90 min, thus making the session more age appropriate. All training sessions were conducted at a hospital location after school hours. In order to ensure further in vivo exposure, the participants were contacted by telephone 2 weeks after the training session. A short assessment was then made of the participant’s experience in participating in the session. In addition, the participants were given prompts for data purposes.

#### Therapists

The sessions were conducted by two clinical psychologists with experience in CBT treatments of social anxiety disorder and received 4 h of protocol-specific training. The same therapist that conducted the session also contacted the participants by telephone after the training session. The therapists were supervised by a senior clinical psychologist throughout the treatment period.

### Measurements

#### Primary outcome measure

The Public Speaking Anxiety Scale (PSAS; [28]) was administered at screening, at the beginning of the session, post-treatment and at 1 and 3-month follow-up. The PSAS covers cognitions, behaviors and physiological manifestations of Public Speaking Anxiety with 17-items, with a five-point Likert response format. Five of the items are reverse coded. The questionnaire was translated into Norwegian for this study according to scientific standard (including back-translation). Cronbach’s alpha at pre-treatment was an acceptable 0.76.

#### Moderators of treatment effect

Two possible moderators of treatment effects were examined. The Social Interaction Anxiety Scale (SIAS; [29]) was administered at screening and measures the more generalized type of SAD which includes interaction anxiety. SIAS is a 20-items scale on a scale from 1 “not at all characteristic or true of me” to 4 “extremely characteristic or true of me”. Summary scores ranges from 20 to 80 with a higher score indicating more social interaction anxiety. SIAS show good psychometric properties, and discriminates patients with social phobia from patients with other anxiety disorders or no disorder at all. Cronbach’s alpha at screening was calculated to 0.86. As the literature distinguishes between the two subgroups of SAD [3], we investigated whether performance-only social anxiety had better treatment outcome than adolescents with the more generalized type of social anxiety, which includes both performance and interaction anxiety. A binary variable corresponding to “low SIAS” (0, reference) and “high SIAS” (1) was created using median-split subsampling (median = 35), creating two groups (n_low_ = ≥35, n_high_ = ≤ 36–100). This binary variable was used to examine moderating effects, since interaction effects were unlikely to be linear.

Sense of presence in VR environment, the degree to which the experience feels real [32], was also explored as a possible moderator of treatment effects. Sense of presence is positively associated with emotional distress in VR [33], yet the causal, possibly bi-directional relationship between presence and emotional distress is complex [34]. In this study, the Gatineau Presence Questionnaire [35] was administered at the end of the session. The Gatineau Presence Questionnaire is a short measure with five items rated on a 0–100 scale resulting in an average score in percentage. The questionnaire assesses (1) the impression of being in the virtual environment, (2) the experience as being real, and the reversed items; (3) attentiveness of the virtual environment as being artificial, (4) the experience of being present in the office instead of the virtual environment and (5) the experience of discomfort. A presence score is calculated by averaging items 1–4. A binary variable “low presence” (0, reference) and “high presence” (1) was then created using median-split subsampling, creating two groups (n_low_ = ≥ 59, n_high_ = ≤ 60. This binary variable was used to examine moderating effects, since interaction effects were unlikely to be linear.

#### Physiological data

Heart rate data was collected continuously during the session using a wearable, wireless Empatica E4 wristband. Timestamped heart rate data were synchronized to the log files of the VR-running smartphone such that for each data point (temporal resolution: 1 s), the virtual scenario in which each data point was recorded was known. In total, *n *= 147,322 data points from *n* = 21 participants were available for analyses. Time spent in the lobby before entering the full classroom served as comparison period for each exposure period, with the first 60 s of data after each exposure period being discarded to allow heart rate normalization. Mean heart rate during each period and task were calculated. The median number of recorded transitions from lobby to full classroom was eight, with a maximum of 14. Since repetitions of and deviations from the seven per-protocol speech exercises was not systematically recorded, it is not possible to assert whether recoded exposure tasks are equivalent across participants. Only the initial eight exercises for each participant were included in analyses since these likely show the least variations across participants.

### Analyses

SPSS Statistics version 24 was used to analyze data. Outcome data were analyzed using linear mixed effects models [36], modeling change on both individual and group level. The analysis included unstructured random effects covariance matrices, random slopes and intercept. All participants who began treatment were included in analyses, with missing data estimated using restricted maximum likelihood modeling of random effects. PSAS scores served as the dependent variable in all models, with a binary independent variable corresponding to before and after treatment (both screening and pre-measurement coded as zero and the post-measurement as one). Moderation analyses were performed using the same time variable, a binary moderator, and the interaction thereof. In the analysis of long-term effects, a separate mixed model was run using a new, numeric time variable corresponding to months since treatment (0 = post, 1 = 1-month follow-up, 3 = 3-months follow-up). Calculation of effect sizes was based on estimated means pre and post treatment: Pre_m_ − Post_m_/SD_pre_ where the calculation of standard deviation = standard error × $\surd {\text{N}}$. Heart rate data were also analyzed using mixed models (random slopes and intercepts), with period-average heart rate as the dependent variable and period (lobby or full classroom) as independent variable.

## Results

### Attrition

A total of 38 participants completed the online screening, 32 participants (84.2%) were invited to participate in the study and to the training. Two participants cancelled the training session due to lack of time, one did not show up at the training session, one participant was referred to other health services and one participant was excluded due to missing consent from parents. This resulted in *N* = 27 participants: *n* = 6 male (22%) and *n* = 21 female (78%). Participants ranged from 13 to 16 years old, with an average age of 14.22 years (*SD* = 0.64). Observed mean pretreatment level and the mean change and standard deviations for the primary outcome variable PSAS are presented in Table 1.

**Table 1:** Observed means, standard deviations and n missing for primary outcome measure (PSAS) at each measure point

Day	Assessment	*M*	*SD*	*n* missing
0	Screening	62.81	9.88	0
7	Pre-treatment	61.04	7.80	0
21	Post-treatment	49.28	10.23	2
42	One-month follow-up	50.00	12.73	1
97	Three-months follow-up	47.25	11.92	3

*M* mean, *SD* standard deviation, *n*: number of participants, *PSAS* Public Speaking Anxiety scale

### Changes from pre-treatment to post-treatment

Table 1 shows observed means and standard deviations for all measure points. PSAS symptoms remained stable from screening to pre-treatment. The unconditional mixed model showed a significant decrease in their PSAS score from pre to post by an average of 12.23 points (*SE* = 2.08, *p* < 0.001). The effect size, calculated from estimated means pre and post treatment and standard error: 61.04 − 48.81/7.98 = 1.53.

### Changes from Post-treatment to Follow-ups

Modeling the follow-up period revealed a non-significant decrease in PSAS score of − 0.44 (*SE* = 0.41, *p* = 0.300) per month after treatment. See Table 1 for observed scores.

### Moderators of treatment effects

Mixed effects models were computed in order to investigate whether there was any difference between groups when exploring moderators of treatment effects, by using low generalized social anxiety and low experience of presence as a reference in the analyses. Moderators of treatment effects showed no difference between groups in treatment outcome from pre to post. See Table 2 for details.

**Table 2:** Estimated treatment effects on primary outcome measures (PSAS)

	*β*	*SE*	*p*	95% CI
Unconditional pre-post				
(Intercept)	61.93	1.56	< 0.001	58.72 to 65.13
Time	− 12.23	2.08	< 0.001	− 16.52 to 7.94
Moderation pre-post by presence (median-split)				
(Intercept)	60.36	2.16	< 0.001	55.91 to 64.81
Presence	2.48	3.18	0.443	− 4.08 to 9.03
Time	− 10.99	3.19	< 0.01	− 17.57 to 4.40
Presence × time	− 4.04	4.60	0.389	− 13.54 to 5.46
Moderation pre-post by generalized SAD (median-split)				
(Intercept)	60.04	2.12	< 0.001	55.67 to 64.40
SIAS	3.93	3.05	0.21	− 2.36 to 10.21
Time	− 14.68	2.82	< 0.01	− 20.51 to − 8.85
SIAS × time	5.10	4.07	0.22	− 3.31 to 13.51

*N* = 27

*Β* (unstandardized) parameter estimates, *SE* standard error, *CI* confidence interval

### Physiological response to exposure

**Fig. 3:**
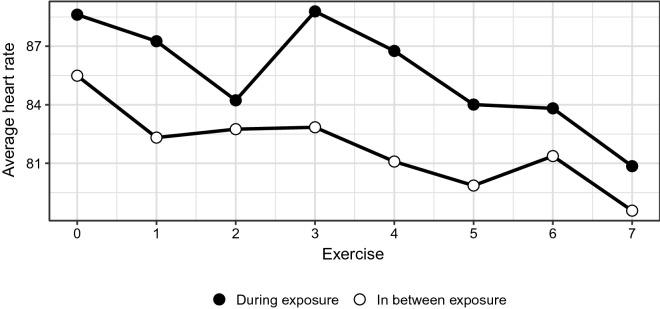
Heart rate

Average heart rate during exposure was 85.89 (*SE* = 1.59), rising on average 3.66 (*SE* = 1.03, *p *< 0.001) from the non-exposure period immediately preceding it. Plotting the data over time revealed that this difference differed substantially between tasks, but because task-equivalence across participants cannot be assumed, it is not possible to draw conclusions as to whether some tasks led to greater increases of heart rate. See Fig. 3.

### Feasibility outcomes

One main purpose of this study was to examine the feasibility of the protocol. Five elements were adjusted from the first (spring 2018) to the second period (autumn 2018) of the study.

#### Parent involvement

Parents were more involved in the second period compared to the first period through e-mail distribution and telephone contact. They were given more detailed information at the beginning of the exposure session, and were asked to ensure that the adolescents fill out the follow- up assessments.

#### Attrition

During the first period, the gift card was distributed to the adolescents after completion of 1-month follow-up, which resulted in difficulties in collecting the 3-month follow-up questionnaire. By adjusting the value of the gift card and distributing them to the adolescents after having completed the 3-month questionnaires, the study did not have any missing data during the second period.

#### Compensation

Due to the workload for the adolescents, we increased the value of the gift card from 200 to 300 NOK (30 euros).

#### Break

Due to the long duration after school hours, a short break was included halfway through the intervention and before starting on the relapse prevention part.

#### Duration of training session

According to the protocol, the intervention was supposed to be 90 min. However, this was not applicable and time spent on each training session was between 2 and 2.5 h.

## Discussion

The present study is the first to examine the feasibility, to pilot the effects of a VRET intervention for adolescents with fear of public speaking. In addition, the aim was to examine moderators of treatment and heart rate during the intervention.

A total of *N* = 27 eligible adolescents between 13 and 16 years participated in the study, the majority being female. The original protocol was developed for adults, and adjusted for this age group. Through feedback from the participating adolescents and the therapists, a further adaption to the target group was conducted during the study period. This included increasing the training session from 90 to 120–150 min with a short break, increasing parent involvement, increasing gift card value and introducing prompts after the treatment session for in vivo exposure and data-collection purposes.

The one-session treatment showed large effects on the primary outcome measure (PSAS, *ES* = 1.53) 1 week after treatment. This result remained stable during the one and three follow-up period. This result is comparable to the findings from previous studies on VRET for PSA for adults [23, 25, 26, 37]. The present study showed a large effect size and a reduction in PSAS scores of 12.23 points, whereas the Lindner et al. [26] study showed a 6.90 point reduction. This difference indicate that the original protocol was successfully adapted before and during the trial to our target group. This includes the development of a VR stimuli tailored specifically to illustrate a cultural and age appropriate classroom and audience.

With regards to treatment moderators, the results revealed no significant differences in symptom reduction on the basis of a high general social anxiety symptoms (SIAS scores). Neither did our results show a moderating effect of high sense of presence. While previous research has shown robust associations between presence and emotional distress during the VR exposure, the role of presence in explaining treatment outcome remains unclear [34, 38]. Of note, both these moderation analyses were low–powered, and should therefore be interpreted with caution.

Results from analyses on physiological data revealed that the exposure scenarios were successful in eliciting a physiological response in the form of increase heart rate, yet the increase was small. This is in line with clinical experience from the Lindner et al. [26] trial, finding that VR public speaking scenarios elicit a weaker fear response compared to e.g. VR spider scenarios [39]. The combination of a relative weak physiological response during treatment and a large decrease in the psychological symptoms is congruent with the theoretical foundation for the treatment protocol, emphasizing inhibitory learning over a strong fear response and subsequent habituation rationale [30]. This is also consistent with empirical findings showing that a strong fear response is not necessary for fear extinction [40].

PSA has been until now difficult to treat in a traditional therapeutic setting, due to the stimuli required. VRET is not a common method in the healthcare services, as VR-devices has not been easily available, and has a high cost. With innovative and accessible consumer technology, VRET for PSA may now be offered as a tool for therapists, and VRET can be easily conducted in any clinical setting. The therapists involved in the feasibility and pilot study had a CBT background with no prior experience with VRET, and were able to conduct the treatment after only a 4-h workshop. This shows how any CBT clinician can use the treatment method after only a small amount of training. Also, studies conducted both before and after the advent of available consumer VR technology, have shown that clinicians see benefits of using VR to conduct exposure therapy and that they are willing to adopt the technology in clinical practice [26, 41, 42].

Importantly, we also replicate the finding that modern, low-cost consumer VR hardware can be used to administer efficacious treatment [26]. Findings from this study shows how low-cost VR can be used in treatment and by using a mobile app the potential for the scalability of the treatment method.

### Strengths and limitations

One major strength of this study is low attrition among adolescents as the study had few missing data. Another major strength of this study is the investigation of physiological data using heart rate as well as collecting the PSAS symptom scores, which gives valuable information on both their subjective and their objective level of discomfort experienced when talking to an audience.

There are also some limitations of this study, which needs to be addressed. No control or comparison groups were included in this feasibility and pilot study. Consequently, we cannot conclude on cause for change during the intervention. For example, a decrease in PSA symptoms can be attributed to less oral presentations at school during the treatment and follow-up period. Moreover, the current design cannot isolate the therapist effect from the VRET effect, as the participants conducted the VRET intervention with guidance from a therapist. Being adjacent to a therapist when doing the exposure tasks might have contributed to the clinical efficacy of the VR intervention. However, the recent randomized controlled trial conducted by Lindner et al. [26] showed a reduction in anxiety symptoms when doing the intervention at home without therapist-guidance. Miloff et al. [39] showed that limited therapeutic guidance in VRET for spider phobia led to reduction in anxiety symptoms, and the results maintained the same at follow-up. This indicates that the VR intervention itself is contributing to the reduction in PSA symptoms. Moreover, the study did not collect data on generalized social anxiety disorder (SAD) after treatment; in retrospect, it would have been valuable to include such data at post and follow-up and explore whether VRET for PSA also has a treatment effect on SAD, as indicated by previous studies [12–14]. Another limitation is the small sample (although well-powered for the expected effect size), and as is typical of clinical trials, it is unknown to what degree findings generalize to non-treatment seekers. Further, it consists of a higher group of female.

Future studies should examine the effectiveness of the VR intervention in a randomized controlled trial, with a larger population for the generalizability of the study, in addition to providing the intervention as self-guided for scalability purposes. There is also a need to explore the limitations and benefits of VR-delivered exposure in a head-to-head comparison of in vivo and VR-delivered exposure for FoPS. Data on real-world public speaking behavior during the follow-up intervention would be an interesting outcome measure in future studies, in order to investigate the transition from the VR-scenario to the real world context. In this regard, a longer follow-up period (e.g. 12 months) would have been useful.

## Conclusion

The feasibility and pilot study shows that one-session VRET is an effective tool for treating adolescents with PSA. By using a mobile application platform and an affordable VR platform, the study shows the great potential of VRET as scalable option for treating PSA in adolescents.

## Data Availability

The datasets used and/or analyzed during the current study are available from the corresponding author on reasonable request.

## Abbreviations

PSA: Public Speaking Anxiety
SAD: social anxiety disorder
VR: virtual reality
VRET: virtual reality exposure therapy
CBT: cognitive behavioral therapy
